# Differentiation of orbital lymphoma and idiopathic orbital inflammatory pseudotumor: combined diagnostic value of conventional MRI and histogram analysis of ADC maps

**DOI:** 10.1186/s12880-018-0246-8

**Published:** 2018-05-02

**Authors:** Jiliang Ren, Ying Yuan, Yingwei Wu, Xiaofeng Tao

**Affiliations:** Department of Radiology, Shanghai Ninth People’s Hospital, Shanghai Jiao Tong University School of Medicine, Shanghai, China

**Keywords:** Orbit, Lymphoma, Inflammatory pseudotumor, Diffusion weighted imaging, Histogram

## Abstract

**Background:**

The overlap of morphological feature and mean ADC value restricted clinical application of MRI in the differential diagnosis of orbital lymphoma and idiopathic orbital inflammatory pseudotumor (IOIP). In this paper, we aimed to retrospectively evaluate the combined diagnostic value of conventional magnetic resonance imaging (MRI) and whole-tumor histogram analysis of apparent diffusion coefficient (ADC) maps in the differentiation of the two lesions.

**Methods:**

In total, 18 patients with orbital lymphoma and 22 patients with IOIP were included, who underwent both conventional MRI and diffusion weighted imaging before treatment. Conventional MRI features and histogram parameters derived from ADC maps, including mean ADC (ADC_mean_), median ADC (ADC_median_), skewness, kurtosis, 10th, 25th, 75th and 90th percentiles of ADC (ADC_10_, ADC_25_, ADC_75_, ADC_90_) were evaluated and compared between orbital lymphoma and IOIP. Multivariate logistic regression analysis was used to identify the most valuable variables for discriminating. Differential model was built upon the selected variables and receiver operating characteristic (ROC) analysis was also performed to determine the differential ability of the model.

**Results:**

Multivariate logistic regression showed ADC_10_ (*P* = 0.023) and involvement of orbit preseptal space (*P* = 0.029) were the most promising indexes in the discrimination of orbital lymphoma and IOIP. The logistic model defined by ADC_10_ and involvement of orbit preseptal space was built, which achieved an AUC of 0.939, with sensitivity of 77.30% and specificity of 94.40%.

**Conclusions:**

Conventional MRI feature of involvement of orbit preseptal space and ADC histogram parameter of ADC_10_ are valuable in differential diagnosis of orbital lymphoma and IOIP.

## Background

Orbital lymphoma and idiopathic orbital inflammatory pseudotumor (IOIP) represent the most common lymphoproliferative disorders affecting the orbit [1], accounting nearly 20% of all orbital mass lesions [1–3]. Most orbital lymphomas are low-grade neoplastic lesions, and the most frequently observed subtype is mucosa-associated lymphoid tissue (MALT) lymphoma [4]. IOIP is a non-infective inflammatory condition, which often manifests borderline morphological characters [1]. Lymphoma is amenable to radiation therapy, and combined chemotherapy for high-grade and disseminated lesions, whereas IOIP responds to steroid therapy or immune-suppressive therapy [3, 4]. Therefore, the discrimination of lymphoma and IOIP is essential for clinical treatment. However, dacryoadenitis and diffuse inflammation subtypes of IOIPs are easily misdiagnosed as lymphoma because of similar clinical and imaging findings [5]. Although fine-needle aspiration is the gold standard for diagnosis, it is often limited in technically challenge in the lesions located in the far posterior orbit [6]. In addition, diffuse lymphocytic infiltrative IOIP, as the most common pathological subtype, could not be easily differentiated pathologically from orbital lymphoma [6].

Previous studies have indicated some MRI features, such as signal intensity on T2W sequence, presence of flow void sign, and degree of contrast enhancement, have the potential to discriminate between lymphoma and IOIP [5, 7, 8]. Nevertheless, overall diagnostic efficacy of morphological features was still limited [9]. In addition, the qualitative assessment of MRI features may be observer-dependent. Diffusion weighted imaging (DWI), with the apparent diffusion coefficient (ADC) value, has been used increasingly in the quantitative discrimination of lymphoma and IOIP [8, 10]. Orbital lymphoma showed lower mean ADC value than IOIP, although the overlap of mean ADC value still existed and restricted clinical application. Additionally, the mean ADC value was usually obtained from manually drawing regions of interest (ROI), with potential measurement sampling error and subjective bias. Furthermore, the mean ADC value could not represent the heterogeneity of the whole tumor. Whole-tumor histogram analysis of the ADC maps could generate several diffusion parameters, which have been shown superior efficacy than mean ADC value in discriminating and grading tumors [11–13]. By selecting volume of interest (VOI) covering the whole tumor, the histogram analysis can decrease the sampling errors [14]. Recently, whole-tumor histogram analysis of ADC maps has been demonstrated to accurately diagnose orbital masses [15, 16]. However these studies included a variety of malignant orbital tumors, such as lymphoma, adenoid cystic carcinoma, and metastases.

Therefore, the purpose of this study was to investigate the independent and combined value of conventional MRI features and whole-tumor histogram parameters derived from ADC maps in the differentiation of orbital lymphoma from IOIP.

## Methods

### Patients

The Institutional Review Board of Shanghai Ninth People’s Hospital approved this retrospective study and the requirement for informed consent was waived. The following criteria were adopted for patient selection: 1) The mass was primary orbital tumor; 2) The patients underwent both conventional MRI and DWI scan before treatment; 3) Masses with short axis ≥ 10 mm; 4) The MR images could be acquired and interpreted. Through a comprehensive search of our institutional medical report database from January 2012 to September 2016, we identified 18 patients with orbital lymphoma (mean age, 67 years; range, 45–94 years) and 22 patients with IOIP (mean age, 54 years; range, 8–76 years). The final diagnoses were made upon histopathological results in 36 patients, and upon response to corticosteroid treatment and minimum 1-year follow-up in 4 patients with presumed IOIPs. 16 cases of lymphomas were MALT lymphomas, and the other 2 cases were diffuse large B-cell lymphomas. Myositis and optic perineuritis subtypes of IOIPs were not included in the present study because they demonstrated characteristic findings on conventional MRI, which were easy to be differentiated from lymphoma. In the current study, the patients with IOIPs included dacryoadenitis subtype (*n* = 17), tumor subtype (*n* = 3), and diffuse inflammation subtypes (*n* = 2).

### MRI examination

All MRI examinations were performed on a 3.0 T scanner (Magnetom Verio 3.0 T; Siemens, Erlangen, Germany) with a 12-channel head coil. The conventional MRI protocol for orbital lesions include axial T1-weigthed images (repetition time [TR]/echo time [TE], 620/9 ms), axial fat-saturated T2-weighted images (TR/TE, 4000/75 ms), coronal T2-weighted images (TR/TE, 4000/108 ms), as well as axial, coronal and sagittal contrast enhanced fat-saturated T1-weighted images (TR/TE, 550/9 ms). For contrast-enhanced T1-weighted image, a standard dosage of 0.1 mmol/kg of gadopentetate dimeglumine (Magnevist; Schering, Berlin, Germany) was administrated.

Before contrast medium administration, diffusion-weighted images in the axial plane were acquired using a single-shot spin echo echo-planar imaging (SS-SE-EPI) sequence. The parameters were used as followings: TR/TE, 4000/100 ms; section thickness, 3 mm; flip angle (FA), 90°; field of view (FOV), 200 × 200 mm; matrix, 384 × 384; *b*, 0 and 700 s/mm^2^.

### Image analysis

Qualitative MRI analysis was performed by two radiologists with 3 and 7 years of experience in head and neck imaging, who were blinded to the clinical information and diagnosis. Consensus between the 2 readers was reached by virtue of an additional reading session. The following characteristics were evaluated and recorded from conventional MR images: 1) laterality (unilateral or bilateral), 2) margin (well-defined or ill-defined), 3) location (intraconal, extraconal, intra and extraconal), 4) signal intensity on T2-weighted image (iso- or hypo-intense; relative to cerebral cortex), 5) degree of contrast enhancement (moderate or significant; relative to normal ocular muscle), 6) involvement of orbit preseptal space (the mass located in or extending to orbit preseptal space) 7) presence of “flow void sign” on T2-weighted image (referring to a signal void of a vessel within the lesions), 8) findings indicative of sinusitis (thickness of paranasal mucosal exceeds 4 mm or fluid level or the presence of a retention cyst at paranasal cavity [17]).

Quantitative evaluation of DWI was performed with FireVoxel software (CAI^2^R, New York University, NY, USA) by two radiologists independently. For each case, volume of interest (VOI) was outlined on all sections where the tumor can be visualized. T2-weighted and contrast-enhanced images were used for reference to avoid necrotic components and surrounding tissues. In addition, it should be noted that ADC measurements might be affected by image distortions due to susceptibility artifacts. Therefore the marginal part of tumor was left out to avoid the influence of these artifacts. In patient with bilateral lesions, the larger lesion was analyzed. After the VOI of the lesion was determined, voxel-based ADC map was calculated with standard monoexponential fit S = S_0*_exp.(−b_*_ADC), in which S refers to the signal intensity with motion probing gradients applied and S_0_ refers to the signal intensity with *b* = 0 s/mm^2^. Then a histogram of ADC values with a bin width of 10^− 3^ mm^2^/s was generated, and concurrently the following histogram parameters were obtained including ADC_mean_, ADC_median_, skewness, and kurtosis. In addition, four cumulative histogram parameters were calculated including the 10th (ADC_10_), 25th (ADC_25_), 75th (ADC_75_), 90th (ADC_90_) percentiles of ADC values. To ensure intra-reader reproducibility, the DWI data was analyzed again with a minimum interval of 1 month. The average of the two measurement results was adopted for statistical analysis.

### Statistical analysis

Univariate analysis was performed for each qualitative and quantitative variable in an attempt to elucidate the ability of each variable for discriminating between lymphoma and IOIP. The *x*^*2*^ testing (the Fisher exact testing where appropriate) was used to compare the frequency distribution of each qualitative MRI feature. Mann–Whitney *U* test was used to compare all ADC histogram parameters. Multivariate logistic regression analysis was used to identify significant independent variables in the differentiation of lymphoma and IOIP. Receiver operating characteristic (ROC) curves were used to assess the diagnostic utility of identified variables and diagnostic models. Intra- and inter-observer variability of ADC histogram analysis was tested by calculating intraclass correlation coefficients (ICC). Intra-observer reproducibility was computed from the two measurements of the first reader. Inter-reader reproducibility was computed from the first measurement of reader 1 and the measurement of reader 2. Agreement was interpreted according to ICC as: < 0.40, poor; 0.41–0.60, moderate; 0.61–0.80, good; ≥0.81, excellent [18]. A *P* level of less than 0.05 was considered as a statistical significance. Statistical analysis was performed using SPSS (SPSS Version 19.0, Chicago, IL, USA) and MedCalc (MedClac Version 11.4, Mariakerke, Belgium).

## Results

Four qualitative variables were found significant in the univariate analysis for differentiating orbital lymphoma from IOIP, including tumor margin, degree of contrast enhancement, involvement of orbit preseptal space and findings suggestive of sinusitis. The characteristics of well-defined margin (*P* = 0.003), moderate degree of contrast enhancement (*P* = 0.007) and involvement of orbit preseptal space (*P* = 0.01) were more frequently seen in orbital lymphomas. Findings suggestive of sinusitis (*P* = 0.031) were more common in IOIPs. The qualitative MRI features of lymphoma and IOIP are listed and compared in Table 1.

**Table 1:** Frequency Distribution of Qualitative MRI Features

Qualitative Feature	Orbital Lymphoma (*n* = 18)	IOIP (*n* = 22)	*P* value
Laterality			0.090
Unilateral	15	12	
Bilateral	3	10	
Margin			0.003
Well-defined	16	9	
Ill-defined	2	13	
Location			0.464
Extraconal	12	17	
Intra and extraconal	6	4	
Intraconal	0	1	
Signal intensity on T2WI			0.125
Iso	12	9	
Hypo	6	13	
Degree of contrast enhancement			0.007
Moderate	10	3	
Significant	8	19	
Involvement of orbit preseptal space			0.01
Yes	13	6	
No	5	16	
Presence of flow void sign on T2WI			0.761
Yes	8	11	
No	10	11	
Findings suggestive of sinusitis			0.031
Yes	5	14	
No	13	8	

The signal intensity on T2WI was compared with that of cerebral cortex. On contrast enhanced T1WI, the similar enhancement to normal ocular muscle was viewed as significant contrast enhancement

**Fig. 1:**
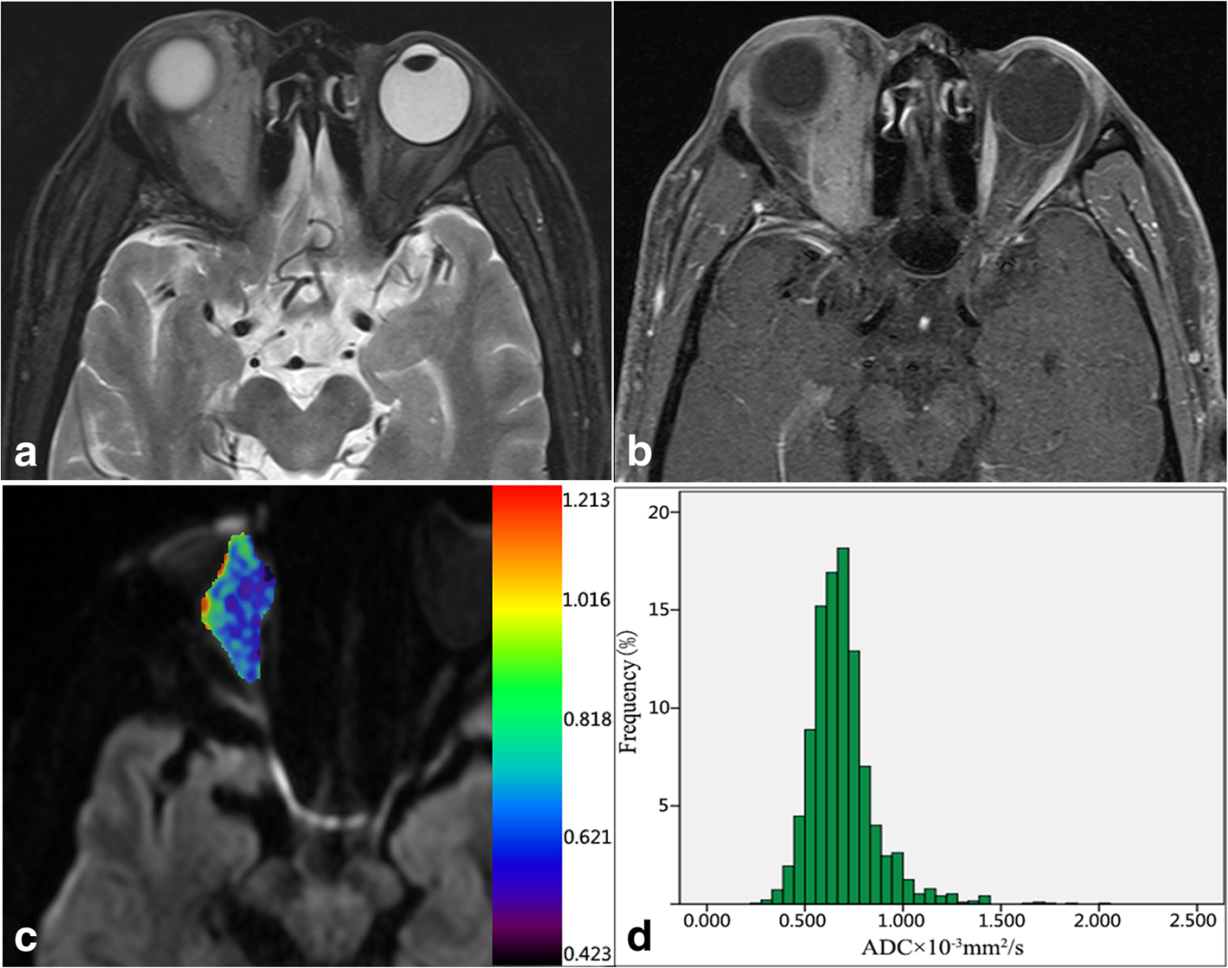
A male patient with MALT lymphoma. Axial fat-saturated T2W image **a** shows a well-defined isointense mass involving intra and extraconal space and extending to the orbit preseptal space. Axial contrast enhanced T1W image **b** demonstrates homogenous moderate enhancement, lower than that of extraocular muscles. Pixel-by-pixel colored ADC map **c** was obtained and then embedded with the axial DWI. The corresponding ADC histogram from VOI **d** shows a top peak and a slight tail on the right. Whole-tumor mean ADC was 0.692 × 10^− 3^ mm^2^/s, ADC_10_ was 0.520 × 10^− 3^ mm^2^/s, skewness was 1.81, and kurtosis was 7.51

**Fig. 2:**
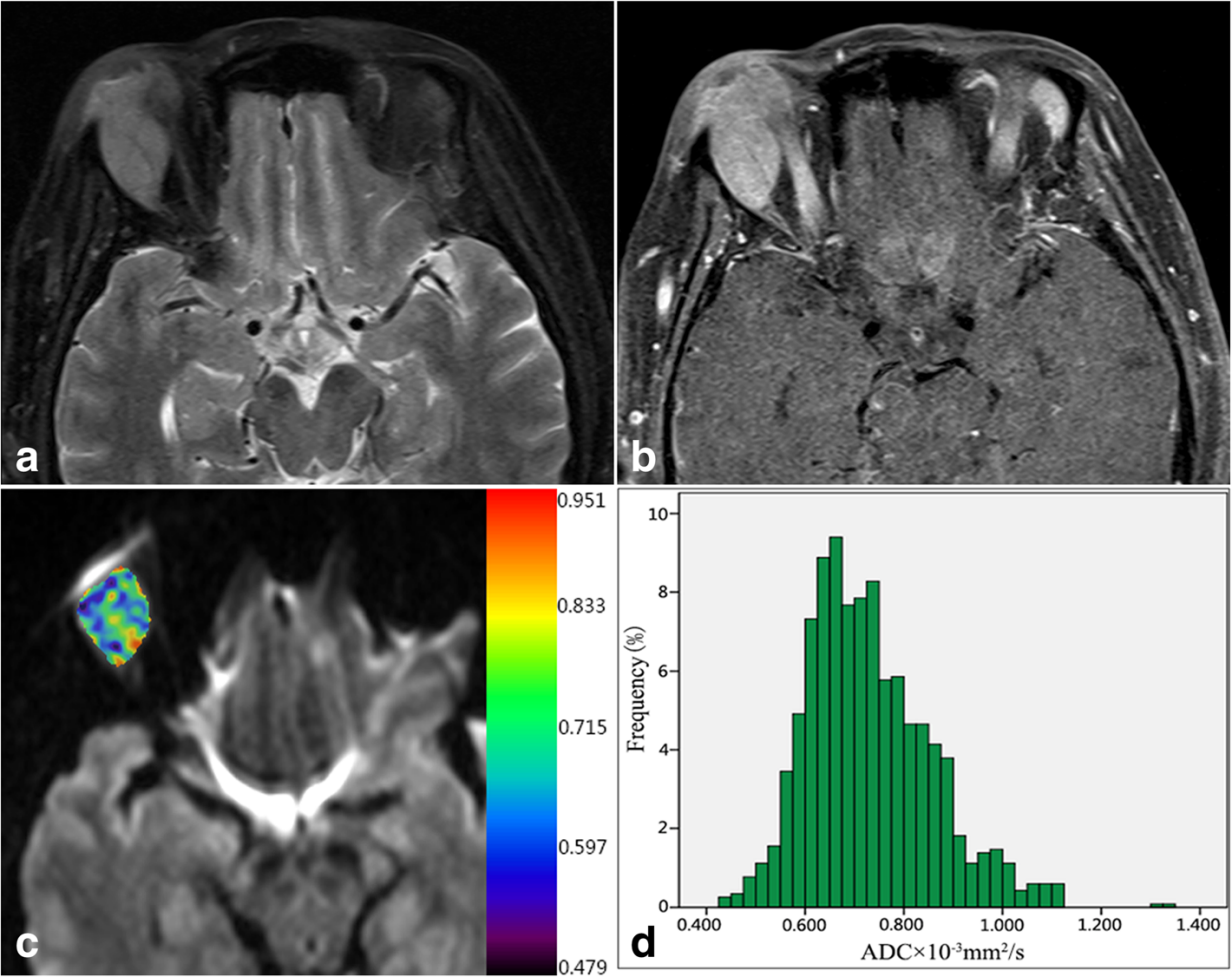
A female patient with idiopathic orbital inflammatory pseudotumor. Axial fat-saturated T2W image **a** shows a well-defined isointense mass in lacrimal fossa. Axial contrast enhanced T1W image **b** demonstrates significant enhancement, similar to that of extraocular muscles. Pixel-by-pixel colored ADC map **c** was obtained and then embedded with the axial DWI. The corresponding ADC histogram from VOI **d** a relatively flat peak and a slight tail on the right. Whole-tumor ADC_mean_ was 0.730 × 10^− 3^ mm^2^/s, ADC_10_ was 0.590 × 10^− 3^ mm^2^/s, skewness was 0.73, and kurtosis was 0.80

Based on the intraclass correlation coefficient (ICC), assessment of intra- and interobserver variability in ADC measurements demonstrated good to excellent agreement with intraobserver ICC values ranging from 0.774 to 0.854 and interobserver ICC values ranging from 0.782 to 0.870, respectively. The comparison of the ADC histogram parameters between lymphoma and IOIP is shown in Table 2. The ADC_mean_, ADC_median_, ADC_10_, ADC_25_, ADC_75_, and ADC_90_ were significantly lower in orbital lymphoma (*P* < 0.001), whereas kurtosis was significantly higher in lymphoma (*P* = 0.023), when compared to IOIP. There was no significant difference in the skewness between the two groups. The representative cases are shown in Figs. 1 and 2. By generating and comparing the ROC curve of each histogram parameter, we found that ADC_10_ achieved the highest area under the curve (AUC) of 0.899 in the differentiation of orbital lymphoma from IOIP. Considering of collinearity between the ADC histogram parameters, only the parameter with the best differential performance was adopted for further multivariate logistic regression analysis. The diagnostic performance of histogram parameters is shown in Table 3.

**Table 2:** Differences of Histogram Parameters between Lymphomas and IOIPs

Histogram parameter	Orbital lymphoma	IOIP	P value
Mean	0.719 ± 0.216	1.131 ± 0.317	< 0.001
Median	0.686 ± 0.202	1.099 ± 0.332	< 0.001
ADC_10_	0.463 ± 0.150	0.794 ± 0.244	< 0.001
ADC_25_	0.562 ± 0.163	0.923 ± 0.275	< 0.001
ADC_75_	0.832 ± 0.269	1.305 ± 0.384	< 0.001
ADC_90_	1.005 ± 0.336	1.519 ± 0.430	< 0.001
Skewness	1.314 ± 1.042	0.820 ± 0.737	0.135
Kurtosis	4.959 ± 6.208	1.829 ± 2.923	0.024

Data are presented as mean ± standard deviation. Unit for ADC value is 10^−3^ mm^2^/s. ADC _n_, nth percentile value of cumulative ADC histogram

**Table 3:** Diagnostic Performance of Each Histogram parameter

Histogram parameter	Cutoff value	Sensitivity	Specitivity	AUC
Mean	0.879	72.70%	94.40%	0.871(0.727–0.956)
Median	0.834	72.70%	94.40%	0.872(0.729–0.957)
ADC_10_	0.535	86.40%	83.30%	0.899(0.762–0.972)
ADC_25_	0.718	72.70%	94.40%	0.895(0.757–0.970)
ADC_75_	1.097	72.70%	94.40%	0.872 (0.729–0.957)
ADC_90_	1.280	68.40%	93.70%	0.851(0.703–0.944)
Kurtosis	1.976	72.70%	72.20%	0.710(0.545–0.842)

Data in parentheses indicate 95% confidence intervals. Unit for ADC value is 10^−3^ mm^2^/s. ADC _n_, nth percentile value of cumulative ADC histogram; AUC, area under the ROC curve

**Fig. 3:**
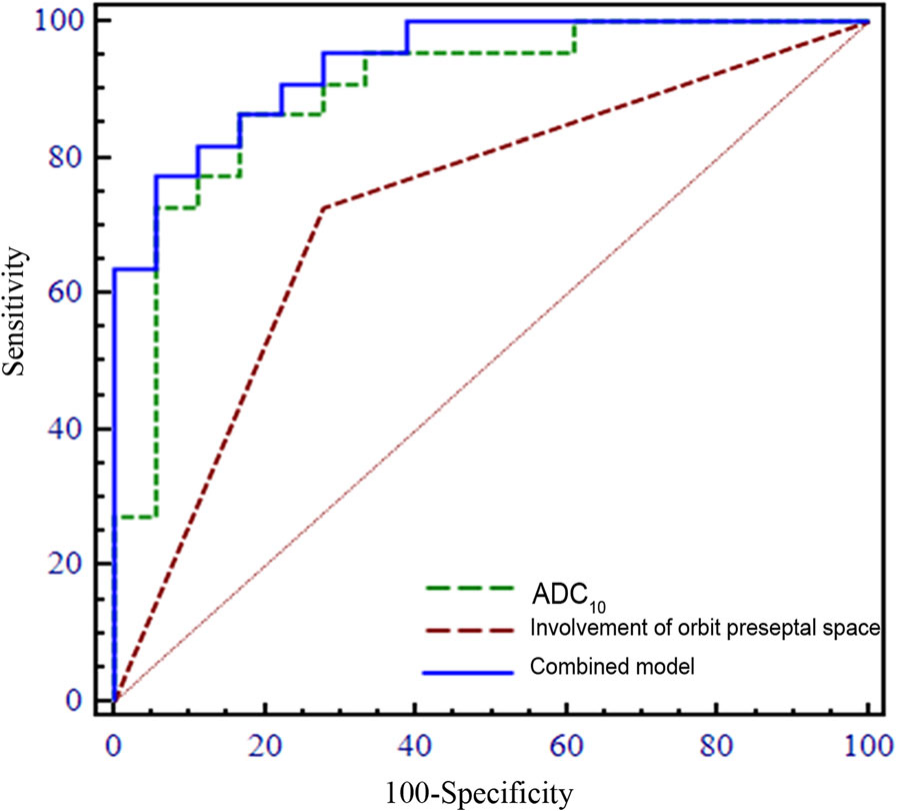
Receiver operating characteristic (ROC) curves of ADC_10_, involvement of orbit preseptal space, and combined model for differentiating orbital lymphomas from IOIPs. Area under the ROC curves (AUCs) of ADC_10_, involvement of orbit preseptal space and combined model were 0.899, 0.725, 0.939, with sensitivity of 86.40%, 72.70%, 77.30% and specitivity of 83.30%, 72.20%, 94.40%, respectively

Histogram and conventional MR parameters, including ADC_10_, margin, degree of contrast enhancement, involvement of orbit preseptal space and findings suggestive of sinusitis, were entered into a multivariate logistic regression model. Multivariate logistic regression identified ADC_10_ (*P* = 0.023) and involvement of orbit preseptal space (*P* = 0.029) as significant independent variables for discrimination. By performing the ROC analysis, we found the combined model (AUC, 0.939; sensitivity, 77.30%; specificity, 94.40%) defined by these two parameters achieved a significantly higher AUC (*P* = 0.0017) than morphological feature of involvement of orbit preseptal space alone (AUC, 0.725; sensitivity, 72.70%; specificity, 72.20%). However, no significant difference of AUC was detected between combined model and ADC_10_ (*P* = 0.294). ROC curves of ADC_10_, involvement of orbit preseptal space and combined model are shown in Fig. 3.

## Discussion

Our primary results demonstrated that involvement of orbit preseptal space and ADC_10_ were the most significant independent variables for discriminating orbital lymphoma from IOIP. To our knowledge, our study was the first to evaluate the combined diagnostic value of conventional MRI and ADC histogram for the differentiation of the two entities.

With respect to conventional MRI features, we found involvement of orbit preseptal space was more frequently observed in orbital lymphomas (13/18) than IOIPs (6/22). Akansel et al. [5] reported nearly half of orbit lymphomas were located in conjunctiva and Giovanni et al. [4] detected that eyelid was involved in 35% of orbital lymphomas. Generally orbital lymphomas are originated from lymphoid tissues, which are confined in eyelid, conjunctiva and lcarimal gland, while IOIPs are in nature inflammatory condition which could arise from elsewhere without lymphoid tissues, such as ocular muscle, orbit fat and optic nerve, without anterior preseptal involvement [3]. Previous studies have shown an extension of inflammatory changes to the mucosa of paranasal cavities in benign orbital lymphoproliferative disorders [8, 9, 19]. We also found that imaging findings indicative of sinusitis were more commonly seen in IOIP (14/22) than orbital lymphoma (5/18). We speculated that both of IOIP and paranasal sinusitis could be the clinical manifestation of idiopathic inflammation involving different structures. In addition, significant enhancement was observed more frequently in IOIP in the current study. Haradome et al. [8] reported that contrast enhancement ratio of IOIP was significantly higher than that of orbital lymphoma, suggesting the hypervascular nature of the former. These qualitative characteristics could provide valuable information for the differentiation of orbital lymphoma and IOIP. Previously some investigators revealed that the presence of flow void sign on T2WI was more easily observed in IOIP than lymphoma [8]. However, we found no significant difference in the presence of a vessel signal void on T2WI between the two entities, which might be attributed to the sample difference and low reproducibility of qualitative MRI evaluation.

Besides morphological MR findings, significantly lower mean ADC value in orbital lymphoma than IOIP has been demonstrated in prior investigations [8, 10, 20]. High cellularity and enlarged nuclei of orbital lymphoma lead to the relative reduction in extracellular and intracellular diffusion spaces and corresponding decreases ADC value. On the other hand, interstitial edematous change in IOIP gives rise to increase ADC, promoting a significant difference in the ADC value than lymphoma. However, mean ADC value ignored the heterogeneity of the tumors. In fact, overlap exists between average ADC value of orbital lymphoma and IOIP [9, 20].

Being capable to measure the distribution frequency of ADC values as a marker of structural heterogeneity and complexity, ADC histogram analysis has been gaining increasing clinical adoption for differential diagnosis of benign and malignant abnormalities [16, 21], and assessment of tumor grade [11, 22, 23]. In the current study, we observed ADC_10_ had a better performance than ADC_mean_ in the differentiation. Previous studies have reported low percentiles of ADC performed better in classification and grading of tumor than high percentiles [21, 24]. Rozenberg et al. [22] suggested that ADC_0–10_ metric achieved greatly superior performance in the differentiation of low- from high-Gleason score prostate tumors. Kierans et al. [24] revealed ADC_10_ allowed for the accurate prediction of malignant endometrial lesions, while ADC_mean_ did not. Recently, Xu et al. [15, 16] reported ADC_10_ could predict malignant orbital tumors with a higher AUC than ADC_mean_. Given our similar results in the differentiation of orbital lymphoma and IOIP, it is possible that low percentiles of ADC truly better reflect the presence of densely packed solid components within tumor tissues. The high percentile of ADC value may be easily vulnerable to the cystic or necrotic components. In clinical, small areas of microcystic changes are usually failed to be excluded from region of interests because of limitation of visual detection, which may affect the accuracy of ADC measurement. Therefore, low percentiles of ADC seemed to be more effective in discriminating the two lesions with distinct compactness.

In addition, we found significantly greater kurtosis in orbital lymphomas than IOIPs. This might be attributed to the homogeneity of orbital lymphomas. Pathologically, orbital lymphoma consists of monomorphous sheets of lymphocyte, which would appear as a steep peak in histogram [7]. In contrast, IOIP often shows polymorphous inflammatory reaction comprising mature lymphocytes, plasma cells, eosinophils and varying amounts of fibrous stroma [7]. However, there was no significant difference in skewness between orbital lymphomas and IOIPs. Both showed positive skewness. Skewness reflects the asymmetry of the histogram distribution. Positive skewness indicates the majority of voxel values accumulating toward the lower end of the histogram [24, 25]. The visual necrotic and cystic areas with relatively higher ADC values were excluded from VOIs, which could interpret positive skewness in both groups.

With the combined qualitative MRI variable and ADC_10_, diagnostic model achieved a higher AUC and specificity than using either alone in the differentiation. The optimal specificity would enhance our diagnostic confidence, which is necessary for the prompt determination of a therapy plan and doctor-patient communication. However, significant difference of AUC between combined model and ADC_10_ was not observed. Further studies with a larger sample size would be needed to validate our results.

Most quantitative DWI studies of orbital tumors have been based on 2-dimensional region of interest (ROI) placed on a representative slice of the tumor [20, 26]. In the present study, we used VOIs that covered the whole lesions. This method could allow comprehensive measurement, avoiding sample bias from selection of a localized area of the tumor [22]. Given that the histogram parameters, such as skewness and kurtosis, reflect the cumulative distribution of the ADC values, it would be rational to use whole-tumor VOIs rather than localized ROIs for analysis. Furthermore, the good to excellent intra and inter-observer reliabilities based on intraclass correlation coefficients indicated the reproducibility of this measurement method, which will facilitate further clinical application of histogram analysis of ADC maps.

Our study had several limitations. First, it was a retrospective study with relatively small amount of patients, which may contribute to the inconspicuous difference in the differential utility between combined model and ADC_10_. Secondly, although cases with severe susceptibility artifacts have been excluded, slight image distortion might still exists in some images imperceptible with naked eye. Further improvement of the imaging quality of DWI would be essential for the study of orbital lesions. Turbo field echo with diffusion-sensitised driven-equilibrium preparation (DSDE-TFE) technique and half-Fourier acquired single-shot turbo spin-echo (HASTE) DWI sequence would be feasible to reduce these artifacts [27, 28]. Thirdly, we adopted *b* value of 700 s/mm^2^ for DWI. In fact, *b* values of 800 or 1000s/mm^2^ was more commonly used in previous orbit studies. The threshold values of ADC histogram parameters obtained in the present study might not be applicable for other studies that used b = 800 or 1000s/mm^2^. Moreover, we did not discuss dynamic contrast enhanced magnetic resonance imaging (DCE-MRI) in the current study, which is approved to be helpful for differential diagnosis of orbital lesions [9]. Only conventional contrast enhanced MR images were available in the present study. Further study combining histogram parameters derived from DWI and DCE-MRI would be worth to conduct.

## Conclusion

ADC_10_ and involvement of orbit preseptal space were significant independent variables for differentiating orbital lymphoma from IOIP. Histogram analysis of ADC maps demonstrates the heterogeneity of the lesions, which can provide additional and valuable information for the diagnosis of the two entities.

## Abbreviations

ADC: Apparent diffusion coefficient
AUC: Area under the ROC curve
DWI: Diffusion weighted imaging
ICC: Intraclass correlation coefficient
IOIP: Idiopathic orbital inflammatory pseudotumor
MALT: Mucosa-associated lymphoid tissue
MRI: Magnetic resonance imaging
ROC: Receiver operating characteristic
ROI: Region of interest
VOI: Volume of interest
